# New insights into the tumor immune microenvironment and immunotherapy of thyroid cancer

**DOI:** 10.3389/fimmu.2026.1699500

**Published:** 2026-01-23

**Authors:** Jia-jia Du, Jian Wang, Kai Ma, Peng Ma

**Affiliations:** Shanxi Bethune Hospital, Shanxi Academy of Medical Sciences, Third Hospital of Shanxi Medical University, Tongji Shanxi Hospital, Taiyuan, China

**Keywords:** immunotherapy, metabolic reprogramming, PD-L1, thyroid cancer, tumor immune microenvironment

## Abstract

Thyroid cancer (TC) is the most common malignant tumor of the endocrine system. Although most cases have a favorable prognosis, some patients may be resistant to treatment or exhibit aggressive behavior. The tumor microenvironment (TME) network, composed of stromal cells, immune cells, vascular cells, and cancer cells, has become a key factor in the development of TC. The TME affects the biological behavior of TC through different immune states. TC cells can suppress antitumor immune response by promoting an immunosuppressive microenvironment, such as through the recruitment of tumor-associated macrophages (TAMs), tumor-associated mast cells (TAMCs), myeloid-derived suppressor cells (MDSCs), tumor-associated neutrophils (TANs), and regulatory T cells (Tregs), among other immunosuppressive cells. They also express negative immune checkpoints such as programmed death ligand 1 (PD-L1) and cytotoxic T lymphocyte-associated protein 4 (CTLA-4), and immunosuppressive enzymes such as indoleamine 2,3-dioxygenase 1 (IDO1). This suggests that immunotherapy may be a promising treatment for TC, especially for patients who do not respond to traditional therapies. This article focuses on the interaction mechanism of cells and molecules in the tumor immune microenvironment (TiME) involved in the occurrence and development of TC and analyzes its potential value as a therapeutic target. In addition, the latest clinical trials related to immunotherapy for TC are summarized.

## Introduction

Thyroid cancer (TC) is the ninth most common malignancy and the most common cancer of the endocrine system worldwide. The incidence of women is significantly higher than that of men, resulting in more than 586,000 new cases and 43,600 deaths each year (1). The currently known types of TC include papillary thyroid cancer (PTC), follicular thyroid cancer (FTC), medullary thyroid cancer (MTC), and anaplastic thyroid cancer (ATC). Differentiated thyroid cancer (DTC), which includes PTC and FTC, accounts for more than 90% of all cases (2). Traditional treatment methods include surgery, radiotherapy, and endocrine suppression therapy; however, the efficacy of each therapy remains limited to varying degrees (3). Over the past decade, with an in-depth understanding of the molecular pathogenesis of TC, significant progress has been made in its treatment. The approval of tyrosine kinase inhibitors (TKIs), including neratinib, sorafenib, and cabozantinib, has had a positive impact on the clinical outcomes of patients with radioactive iodine-refractory (RAIR) TC. Subsequently, selective RET inhibitors (selpetinib and palasertinib) and tropomyosin receptor kinase (TRK) inhibitors (larotinib and entrutinib) were developed for patients with advanced metastatic TC harboring RET and TRK fusion genes, respectively. In addition, combination therapy with RET inhibitors and BRAF–MEK inhibitors has also shown efficacy in the treatment of MTC and ATC (4). Innovative therapies such as immune cell-targeted therapy, cancer vaccines, adoptive immunotherapy, and immune checkpoint blockade have significantly improved the efficacy of TC immunotherapy (5). However, due to the lack of a well-defined immune activation mechanism and the unclear mechanism of action underlying different immunotherapy combination strategies, many patients fail to respond to immunotherapy or develop drug resistance (6).

The tumor immune microenvironment (TiME) of TC is composed of a variety of immune cells, including T cells, natural killer cells (NKs), mast cells (MCs), tumor-associated macrophages (TAMs), dendritic cells (DCs), myeloid-derived suppressor cells (MDSCs), and secreted immune factors such as chemokines. These immune cells and factors play a key role in the initiation and progression of TC (7). In addition, cancer-associated fibroblasts (CAFs) promote cell proliferation and extracellular matrix (ECM) remodeling by increasing the expression of epithelial–mesenchymal transition (EMT) marker vimentin while decreasing the expression of the anti-EMT marker E-cadherin in TC cells (8). The activation of immune checkpoints, such as programmed cell death receptor 1/programmed death ligand 1 (PD-1/PD-L1) axis, cytotoxic T lymphocyte-associated protein 4 (CTLA-4), T-cell immune receptor with Ig and ITIM domains (TIGIT), and T-cell immunoglobulin domain and mucin-domain-containing protein 3 (TIM-3), plays a significant role in the immune escape of TC cells, directly promoting tumor cell proliferation and migration (9). In addition, metabolic reprogramming is a key regulator of tumor immune function and contributes to TC initiation and progression. Specifically, amino acid, sugar, and lipid metabolism can influence tumor cell behavior, including proliferation, recurrence, and metastasis, enhancing tumor aggressiveness and mediating treatment resistance. On the other hand, metabolic reprogramming of tumor cells and their metabolites can directly or indirectly affect the normal function of immune cells in the tumor microenvironment (TME), weaken their antitumor immune response, and thereby play an important role in the occurrence and development of TC (10).

This review mainly focuses on the interaction mechanisms between the cellular and molecular components of the TME and their impact on cancer biology. It also discusses the potential of inhibiting the interaction between the TME and tumor cells as a new treatment strategy, aiming to improve therapeutic efficacy and survival rates, as well as to develop new predictive biomarkers.

## Cellular microenvironment

**Figure 1:**
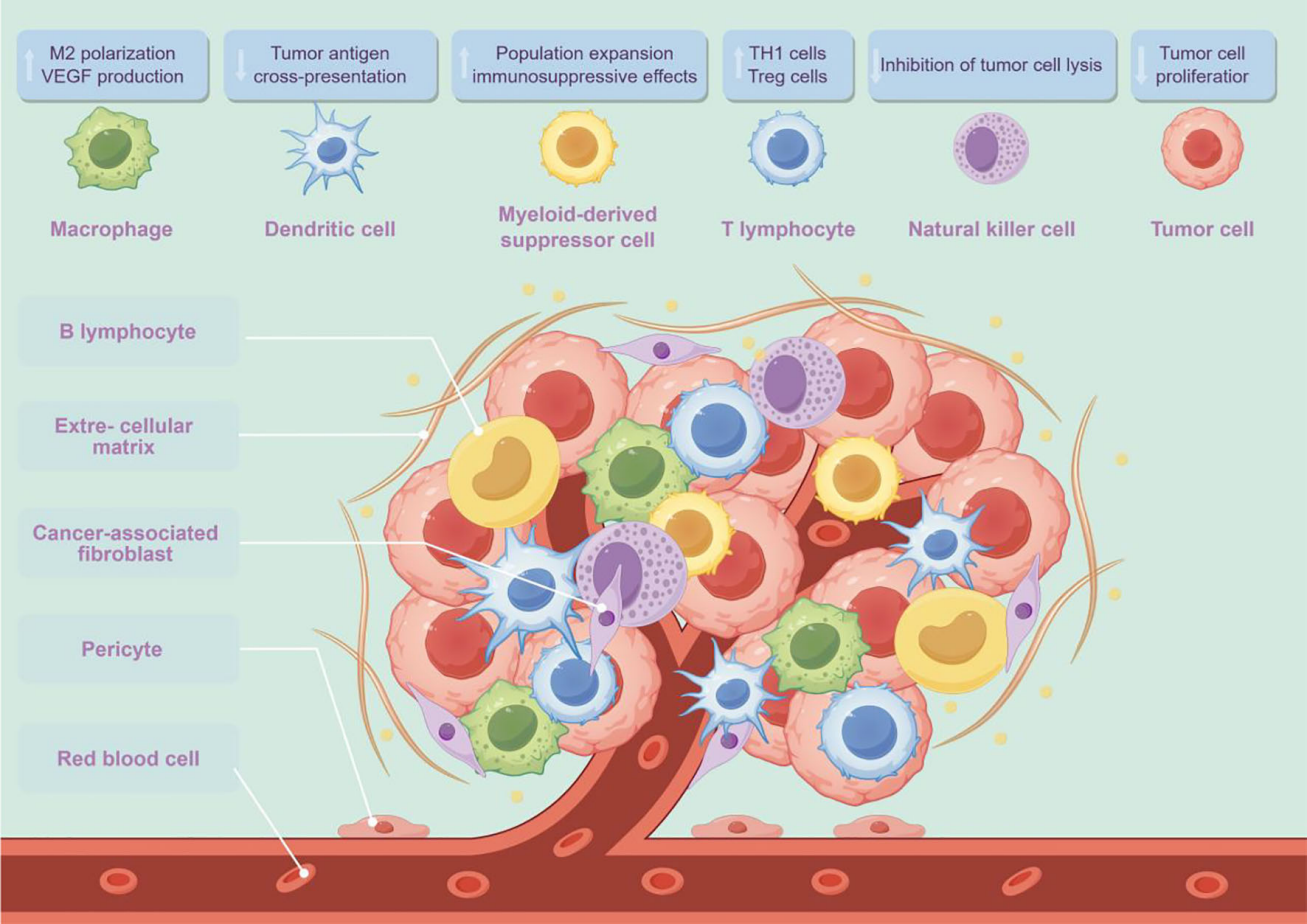
The cellular microenvironment of thyroid cancer (drawn using Figdraw; www.figdraw.com). VEGF, vascular endothelial growth factor; Treg, regulatory T cell.

The TME of TC includes tumor cells and their surrounding milieu, comprising immune cells, stromal cells, and blood vessels (**Figure 1**). These components play key roles in the initiation and progression of TC, and their quantity and proportion vary with changes in host immune status (11). The TME differs significantly among TC subtypes: DTC subtypes have higher numbers of tumor-associated lymphocytes (TALs) and regulatory T cells (Tregs), whereas ATC and MTC subtypes exhibit a higher density of TAMs. One study reported a significant decrease in the proportion of B cells, T cells (predominantly CD8^+^ T cells), and M1 macrophages in the thyroid tissue of patients with PTC compared with healthy controls (12).

### Natural killer cells

NKs have antitumor functions by directly killing tumor cells through granzyme B and perforin. Two subtypes of NKs have been identified: CD56^dim^CD16^+^ NKs with typical cytotoxic functions and CD56^bright^CD16^−/low^ NKs, which exhibit weak cytotoxicity and exert immunomodulatory effects mainly by secreting interleukin (IL)-13 (13).

Compared with healthy individuals, aberrant CD56^bright^CD16^−/low^ NKs are significantly increased in patients with PTC (14). Park et al. noted that PGE2 was highly expressed in PTC. PGE2 secreted by tumor cells inhibits the expression of NK-activating receptors by blocking EP2 and EP4 receptors on the surface of NKs, which in turn suppresses the functional maturation and cytotoxic effects of NKs. Therapeutic strategies targeting PGE2 in TC can enhance the immune response and improve patient prognosis (15).

In addition, the frequency of CD56^hi^CD16^hi/lo^ NKs is significantly increased in ATC patients. CD56^hi^CD16^hi/lo^ NKs not only exhibit significantly reduced cytotoxicity but also highly express exhaustion markers such as PD-1 and TIM-3. Blockade of PD-1 and TIM-3 was found to restore the cytotoxicity of CD56^hi^CD16^hi/lo^ and CD56^lo^CD16^hi^ NKs in ATC patients, suggesting that NKs may be a potential therapeutic target for advanced TC (16). In fact, NKs have been successfully targeted to metastases in preclinical models of lung metastases from ATC, suggesting that NK cell-based immunotherapy may be an effective treatment for ATC (17).

### T lymphocytes

Based on functional differences, tumor-infiltrating T cells can be classified as helper T cells (Ths), cytotoxic T cells (CTLs), and Tregs.

Naive CD4^+^T cells, also known as Th0 cells, can differentiate into Th1 (antitumor), Th2 (protumor), and Th17 subtypes with different immune functions through antigen stimulation and cytokine regulation. The Th1/Th2 ratio is an important indicator for evaluating dynamic changes in antitumor immunity. Th17 cells may play either pro- or antitumor roles depending on the microenvironment. In TC tissues, Th17 levels were significantly higher than in healthy thyroid tissues, a difference also observed in the peripheral blood of patients. Th17 cell levels are inversely correlated with tumor size (18).

The main function of CD8^+^ CTLs is to specifically recognize endogenous antigen peptide–MHC I complexes and subsequently kill tumor cells. This mechanism has become a key indicator for evaluating the prognosis of PTC (19). Patients with higher CD8^+^ CTL expression generally exhibit milder tumor stages and higher survival rates. CD8^+^ T cells exert their protective effect on PTC by recognizing tumor cell surface antigens (20). Bioinformatics studies have shown that the *CLDN10* gene is associated with CD8^+^ T-cell infiltration in PTC and is associated with a favorable prognosis (21).

Tregs are involved in suppressing the immune response in various cancers, thereby promoting disease progression and lymph node metastasis (9). Professor Liu’s team showed that, compared with patients with multinodular goiter (MNG), the proportion of Tregs in peripheral blood and tumor tissues of PTC patients was increased, which correlated with tumor invasiveness. A compensatory increase of Tregs was also observed in the inflammatory sites of PTC tissues with Hashimoto’s thyroiditis (HT), which may lead to suppression of the immune response (22). In addition, Tregs were significantly elevated in the peripheral blood of PTC patients compared with normal thyroid tissues and patients with thyroid adenoma (23).

### B lymphocytes

B lymphocytes are usually present in primary PTC and accumulate in follicular structures in the peritumoral area. They present tumor antigens to tumor-specific T cells (24). By comparing the infiltration status of patients with and without lymph node metastasis, Yang et al. reported a significant negative correlation between B-cell infiltration and lymph node metastasis, suggesting that these immune cells exert a protective effect in PTC (25).

### Tumor-associated mast cells

Tumor-associated mast cells (TAMCs) are widely present in various types of TC tissues, and increased TANC density is associated with greater TC invasiveness. When activated by TC cells, TAMCs produce a variety of soluble factors (such as histamine, IL-6, IL-1, and tumor necrosis factor [TNF] -α). First, TAMCs can promote TC cells proliferation by producing histamine, which binds to H1 and H2 receptors on PTC cells. Second, TAMCs induce EMT and stem cell-like characteristics in human TC cells through the IL-8–Akt–Slug pathway, and blocking this pathway may help treat advanced TC (26).

### Tumor-associated macrophages

Depending on their functional differences, TAMs can be divided into classically activated M1 macrophages and alternatively activated M2 macrophages (27). In TC with Graves’ disease (GD), activated NKs promote the differentiation of macrophages into M1/killer phenotype, which in turn can produce toxic effects on cancer cells and down-regulate M2/repair phenotype (28). Factors secreted by ATC cells induce the polarization of monocytes to the M2 phenotype through a TIM-3-dependent mechanism (29).

TAMs are the most abundant immune cells in the TME of TC, predominantly in the M2-polarized state, providing a favorable microenvironment for tumor growth, survival, and angiogenesis. High TAM density is associated with poor prognosis in TC (30). TAM density also varies by histological subtype; ATC exhibits the highest TAM density and worst prognosis (31). Genes expressed by M2 macrophages, including *FZD6*, *RBBP8*, *PREX1*, and *HSD3B7*, are prognostic factors and are linked to ATC proliferation and invasion (32). Recent studies have identified IL2RA^+^VSIG4^+^ TAMs as ATC-specific subsets with bifunctional M1 and M2 phenotypes, associated with high lymphocyte infiltration and better prognosis (33).

Lv et al. found that M2-type TAMs in PTC could act on the Wnt/β-catenin pathway by increasing the secretion of Wnt1 and Wnt3a *in vitro*, thereby promoting the dedifferentiation, proliferation, and metastasis of PTC (34). In addition, M2-type TAMs promoted IL-6 production and enhanced PD-L1 expression in PTC through the synergistic effect of activating MAPK and JAK-STAT3 signaling pathways, ultimately increasing the invasive capacity of TC (35).

Ahn et al. showed that, in RAIR DTC patients treated with sorafenib, those with a high lymphocyte/monocyte ratio (LMR) had superior overall survival (OS) and progression-free survival (PFS) compared with patients with low LMR. As a result, LMR may serve as a prognostic biomarker for RAIR DTC patients (36). LMR also reflects host immune function and the infiltration of TAMs in PTC, with patients exhibiting low LMR showing lower survival rates (37).

### Tumor-associated dendritic cells

Tumor-associated dendritic cells (TADCs) are the most potent antigen-presenting cells in the human body. Cancer cells act on TADCs by secreting cytokines, thereby affecting their ability to respond to tumor immune challenges (38). TADCs often exhibit an immature phenotype and are unable to fully activate T cells. Although immature TADCs have strong antigen-processing capacity, their ability to promote immune responses is limited, and they may even suppress immunity and facilitate PTC development by secreting inhibitory cytokines such as IL-10 and tumor growth factor β (TGF-β) (20).

Under normal circumstances, TADCs are virtually absent in thyroid tissue. However, their numbers are significantly increased in human PTC tissues, especially CD1a-positive TADCs, which are strongly associated with prolonged disease-free survival (DFS) (39). TADCs highly express thyroid-stimulating hormone (TSH)α and TSHβ2, which can promote tumor cell proliferation, invasion, and immune evasion (40). Higher concentrations of TADCs are associated with more aggressive PTC (41).

Tregs and TADCs can cooperate in immune regulation within the TME. In PTC tissues, Tregs can inhibit TADC function, the expression of costimulatory ligands, and the activation of CD8^+^ T cells. Conversely, TADCs can restore their functions by blocking the PD-1 pathway, inhibiting IL-10 secretion, and reducing lactate production (42).

### Tumor-associated neutrophils

The role of tumor-associated neutrophils (TANs) in cancer is controversial. On one hand, TANs can kill tumor cells, stimulate T cell-dependent antitumor responses, and inhibit angiogenesis (43); on the other hand, TANs may promote genetic instability in cancer cells and release cytokines (such as tumor statin-M and VEGF-A) or granular proteins (e.g., neutrophil elastase) that enhance cancer cell proliferation, invasion, and angiogenesis (44).

In PTC tissues, increased TAN density is positively correlated with tumor volume. Tumor cells recruit TANs by releasing CXCL8/IL-8 and secrete granulocyte colony-stimulating factor (GM-CSF) to reduce their apoptotic rate (45). In addition, Cristinziano et al. found that ATC cells can induce the release of neutrophil extracellular DNA traps (NETs) by TANs through soluble mediators such as CXCL8/IL-8 and reactive oxygen species (ROS). This network structure, composed of cytoplasmic solutes and granular proteins, can effectively promote cancer cell proliferation (46).

Studies have found that an elevated neutrophil–lymphocyte ratio (NLR) is associated with poor treatment response in DTC, suggesting that exacerbated systemic inflammation after treatment may lead to reduced therapeutic efficacy (47).

### Myeloid-derived suppressor cells

MDSCs are a subtype of myeloid cells that exert immunosuppressive functions through ROS, arginin-1, nitric oxide (NO), IL-10, TGF-β, cyclooxygenase 2 (COX-2), and PD-L1 pathways, and are usually associated with poor prognosis in ATC. Compared with healthy controls, circulating MDSCs are significantly increased in patients with ATC, and this increase is correlated with the increase in serum IL-10 level (48). In addition, among ATC patients, the number of tumor-infiltrating MDSCs in long-term survivors (LTS) who have survived for more than 2 years is lower than that in the control group (49).

Circulating MDSCs enhance the activity of the nuclear factor-kappa B (NF-κB) signaling pathway by inhibiting miR-486-3p, thereby accelerating the invasion process of PTC (50). In addition, PTC promotes TC development by increasing the expression level of MDSCs through the BRAFV600E–TBX3–CXCL–MDSC axis (51).

### Cancer-associated fibroblasts

TC is often accompanied by an abundant fibrous matrix, especially in PTC and thyroid adenocarcinoma (52), suggesting the presence of a large number of CAFs. *In vitro* studies have shown that CAF-conditioned medium can increase the expression of EMT marker vimentin and reduce the expression of anti-EMT marker E-cadherin in TC cells, thereby enhancing invasive activity. In addition, CAFs can induce metabolic reprogramming in TC cells, leading to increased glucose uptake and enhanced glycolytic processes (53). In addition, abnormal proliferation of CAFs and extracellular matrix can promote tissue fibrosis and mesothelioma formation, thus constructing a mechanical barrier to prevent the body from producing an effective immune response. In TC, the incidence of tissue fibrosis is directly related to the degree of disease aggressiveness (54).

Studies have shown that a higher concentration of CAFs in patients with TC is associated with an increased risk of lymph node metastasis, shorter OS, and a higher likelihood of harboring the BRAF V600E gene mutation (55). In addition, CAFs can promote the proliferation of monocytes and activated DCs and upregulate the expression of multiple immune checkpoints (such as PD-L1, PD-L2, IDO-1, and CTLA-4) (12). Analysis of CAF-related gene characteristics based on public TC datasets indicates that a high CAF score is positively correlated with dedifferentiation and poor prognosis, suggesting that and targeting CAFs represent a therapeutic strategy for patients with dedifferentiated thyroid cancer (DDTC) (56). Maternally expressed gene 3 (MEG3) is a long noncoding RNA that plays an important tumor-suppressive role in cancer. Dadafarin et al. reported that MEG3 expression levels in PTC can indicate the extent of CAF infiltration (57).

## Immune checkpoints

**Figure 2:**
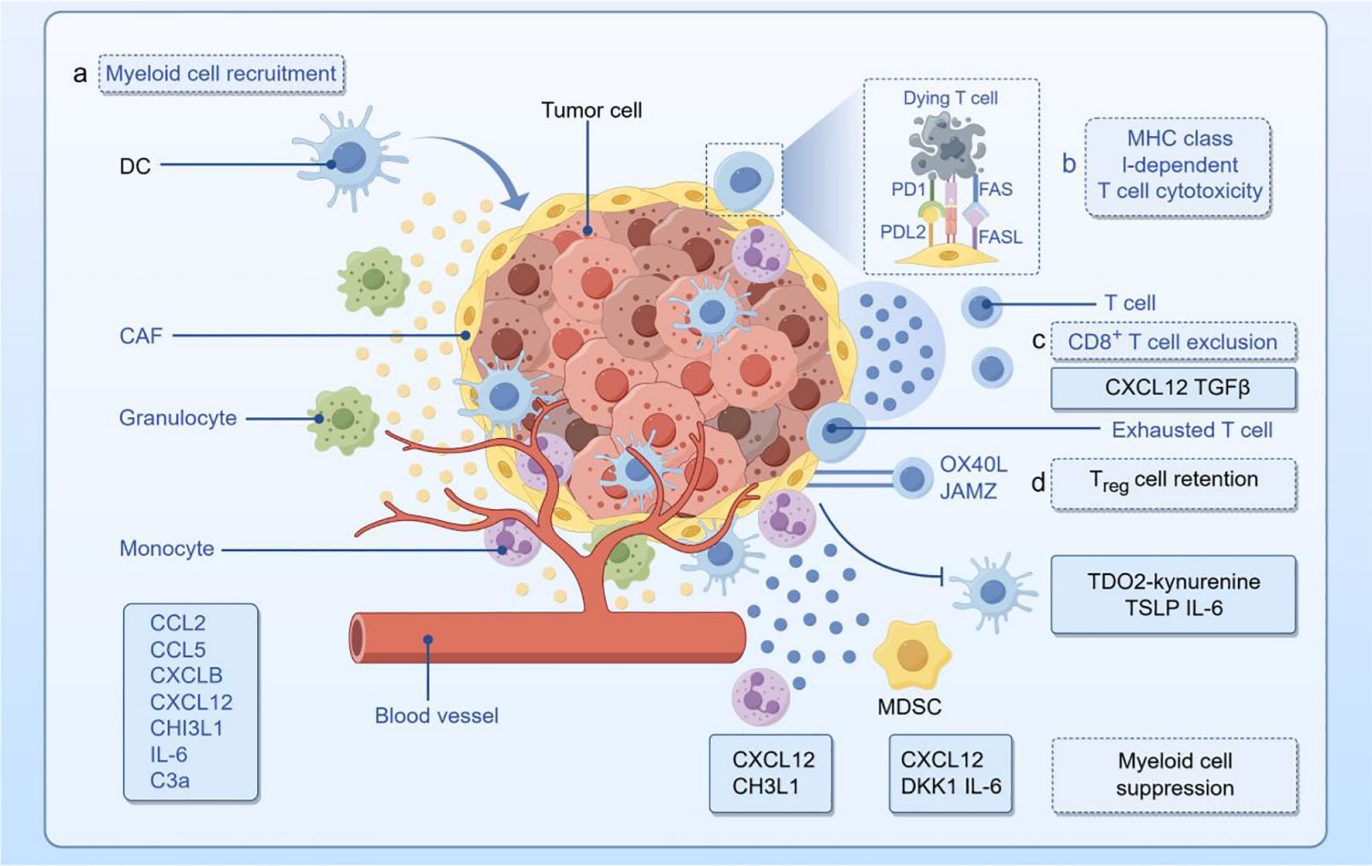
The tumor immune microenvironment of thyroid cancer (drawn using Figdraw; www.figdraw.com). DC, dendritic cells; CAF, cancer-associated fibroblasts; IL-6, interleukin-6; PD-1, programmed cell death protein 1; PD-L2, programmed cell death ligand 2; MHC, major histocompatibility complex; TGF-β, transforming growth factor-β; Treg, regulatory T cell; MDSC, myeloid-derived suppressor cells.

The activation of lymphocytes mainly depends on the recognition of specific antigens by antigen receptors, and the strength, duration, and nature of activation signals are usually regulated by cell surface receptor molecules. As regulatory components, immune checkpoints are responsible for controlling the timing and strength of immune responses, maintaining self-tolerance, and preventing immune hyperactivity. In the TME, these regulators suppress the immune response, resulting in an inability of the body to effectively fight cancer and thereby promoting immune escape (**Figure 2**).

Recent studies have shown that the expression of key immune checkpoints, such as lymphocyte activation gene-3 (LAG-3), PD-1, and IDO1, is suppressed in early-stage PTC compared with normal thyroid tissue, which may be related to preventing immune cells from causing damage to healthy thyroid tissue. However, in advanced stages, the expression levels of most immune checkpoints are significantly increased (12). Giannini et al. showed that in ATC, the expression of inhibitory immune checkpoint mediators CD86, CTLA-4, PD-L1/PD-L2, PD-1, TIGIT, LAG-3, and TIM-3, as well as stimulatory immune checkpoint mediators glucocorticoid-induced tumor necrosis factor receptor (GITR), tumor necrosis factor receptor superfamily member 9 (TNFRSF9), tumor necrosis factor receptor superfamily member 4 (TNFRSF4), and CD40, was upregulated. However, the PTC subgroup showed a lower degree of up-regulation, and poorly differentiated thyroid cancers (PDTC) do not appear to express all of these immune checkpoint mediators (58).

### Programmed cell death protein 1/programmed cell death ligand 1

The PD-1/PD-L1 signaling pathway has emerged as an important inhibitory regulator in cancer. PD-L1 is overexpressed in both DTC and ATC, leading to immune escape (59). The positive rate of PD-L1 in ATC is significantly higher than that in DTC and PDTC (60). PD-L1 expression is present in a subset of PDTC cases (6.1% to 87.5%), and its expression in PDTC is significantly associated with tumor size, multifocality, and poor prognosis (61). Multiple studies have shown a positive correlation between PD-L1 expression levels and the BRAF V600E mutation (62).

### Cytotoxic T lymphocyte antigen-4

CTLA-4 is a transmembrane protein involved in immune regulation. It is usually present on the surface of activated T cells and competitively binds to CD80 and CD86 on the surface of antigen-presenting cells (APCs), thereby competing with CD28. By blocking the involvement of these ligands in CD28 costimulatory signaling, CTLA-4 acts as a negative regulator of T-cell activation (63).

In PTC, the interaction between PD-L1 molecules on the surface of tumor cells and PD-1 molecules on the surface of CTLs leads to CTL apoptosis, which in turn promotes cell proliferation and distant metastasis (64). ATC, for example, shows a limited response to PD-1 inhibitor treatment, possibly because tumor cells contain insufficient CTLs despite being PD-L1 positive. Other studies have indicated that, in most ATC cases, low or absent PD-1 expression on the surface of CTLs limits the therapeutic efficacy of immune checkpoint inhibitors (65). However, animal model studies have shown that preconditioning the immune system by inducing chronic thyroiditis can effectively modulate TC responses to immune checkpoint inhibitors (ICIs) and increase the number of PD-1^+^CTLs in the TiME (66).

### T-cell immunoglobulin domain and mucin domain-3

TIM-3 expression was detected in 48% of MTC. TIM-3 expression is associated with extensive local metastasis, advanced disease, and an increased risk of recurrence (67). TIM-3 regulates cytokine and cytokine receptor expression through the NF-κB signaling pathway and mediates the production and degradation of cell adhesion molecules, thus playing a key role in MTC development (68). Notably, TIM-3 is expressed only on the surface of tumor cells; the TIM-3 protein is scarcely expressed by tumor-infiltrating lymphocytes (TILs) in MTC (69).

### B7 homologous 3

B7 homologous 3 (B7-H3; CD276) is a recently discovered immune checkpoint molecule that inhibits T-cell activation and proliferation and is overexpressed in MTC. Studies have shown that B7-H3 can promote tumor immune evasion and suppress T-cell activity by altering the secretion level of proinflammatory cytokines. Although B7-H3 expression in MTC tumor is approximately three times higher than in normal tissues, its correlation with histopathological features and prognosis remains controversial (70).

## Soluble mediators

In the immune network associated with TC, soluble mediators primarily include substances secreted by tumor-infiltrating immune cells and components produced by TC cells, such as cytokines, chemokines and their signaling pathways, angiogenesis factors, and metabolism-related enzymes.

### Cytokines

Cytokines are small-molecule proteins with a variety of biological activities, synthesized and secreted by both immune cells and nonimmune cells. They play dual roles in the TME, potentially promoting or inhibiting tumorigenesis (71). According to their functional differences, cytokines can be divided into ILs, interferons (IFNs), colony-stimulating factors (CSFs), TNFs, and chemokines.

Several studies have shown that ILs can influence the proliferation of TC cells (71). IL-1α is thought to have protumor activity, promoting tumor differentiation and lymphangiogenesis, whereas IL-1β has been confirmed to inhibit tumor cell proliferation (72). In addition, treatment with the proinflammatory cytokine IL-12, which exhibits potent antitumor activity, significantly reduced tumor volume and weight and prolonged survival in a BRAF V600E-induced PTC mouse model (73). Cunha et al. found that IL-10 expression was positively correlated with extrathyroidal invasion and increased tumor volume, suggesting an important role in TC progression and aggressiveness (74). Activation of the IL-6/JAK2/STAT3 pathway can promote PTC cell proliferation and migration (75). IL-34 promotes proliferation, the EMT phenotype, and extracellular signal-regulated kinase (ERK) signaling, while inhibiting apoptosis in PTC cells (76).

Chemokines are a class of small molecules with chemoattractant and cytokine-like functions, divided into four subfamilies: CXC, CC, C, and CX3C. CXCL8, CXCL12, CCL2, CCL5, and CX3CL1 are highly expressed by PTC and ATC cells and are associated with tumor invasiveness and lymph node metastasis (77).

### Angiogenic factors

Both PTC and ATC express vascular endothelial growth factor (VEGF) (78). VEGF secreted by TC cells can promote neovascularization and inhibit DCs (79). VEGF-A released by TC cells can recruit MCs, a phenomenon closely associated with the aggressive tumor phenotype (80). Elevated VEGF levels can activate the ERK and p38 MAPK pathways and stimulate the production of neuropilin-2 (NRP-2) in TC, thereby increasing susceptibility to lymph node metastasis (81). One study reported a significant positive correlation between VEGF expression and BRAF V600E mutation expression in PTC with extrathyroidal invasion (82).

In addition, VEGF-A, colony-stimulating factor 1 (CSF-1), and cytochemokine 2 (CCL2) attract monocytes into the TME and promote their differentiation into TAMs (83).

### Enzymes

Metabolites are essential for the survival of antitumor immune cells. TC cells can produce metabolic enzymes such as indoleamine 2,3-dioxygenase 1 (IDO1) or arginase-1 (ARG-1), which affect immune cell activity.

IDO1 is an immunosuppressive enzyme. It has been shown that 31% of papillary thyroid microcarcinomas (PTMCs) express IDO1, that IDO1 expression significantly correlates with reduced CD3^+^ TILs and increased FOXP3^+^ TILs, and that IDO1 expression is an independent predictor of extrathyroidal extension and tumor multifocality (84). In addition, the IDO1 pathway inhibits the expression of activating receptors (NKG2D and Nkp46) on the surface of NKs, thereby reducing their number and activity and enabling TC to evade immune recognition (85). ARG-1 hydrolyzes l-arginine to l-ornithine and urea in the hepatic urea cycle. Insufficient concentrations of l-arginine can inhibit T-cell activation and proliferation. Elevated arginase activity has been detected in TC (86). Moreover, IDO1 and ARG-1 secreted by TC inhibit the expression of activating receptors on NKs, resulting in reduced NK cell number and function (7).

In TC, increased COX-2 expression promotes tumor cell invasion and lymph node metastasis (87). In addition, COX-2 impairs tumor immune function by reducing the cytotoxic activity of CD8^+^ T cells, contributing to poor prognosis in patients with DTC (88).

Zhu et al. reported that methyltransferase-like protein 3 (METTL3) enhances STEAP2 expression through an m6A-YTHDF1-dependent mechanism, thereby inhibiting the Hedgehog signaling pathway and EMT to prevent proliferation and metastasis of PTC cells (89). However, another study on METTL3 demonstrated that METTL3 increases the expression of miR-222-3p by accelerating the m6A modification of pri-miR-222-3p; overexpression of miR-222-3p decreased the expression of serine/threonine kinase 4 (STK4) while enhancing proliferation, colony formation, migration, and invasion of PTC cells, ultimately resulting in poorer prognosis for patients (90).

## Metabolic reprogramming

**Figure 3:**
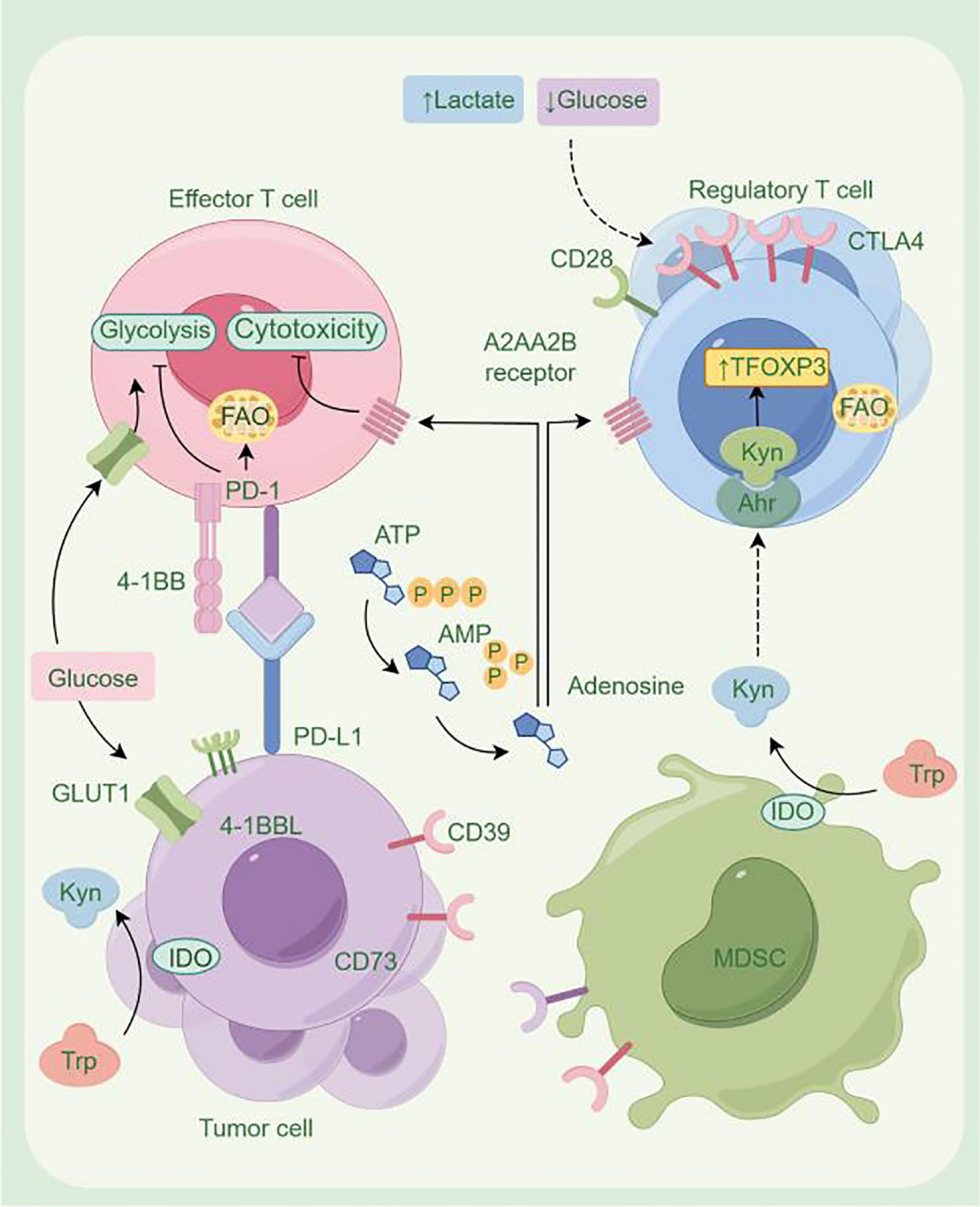
The metabolic reprogramming of thyroid cancer (drawn using Figdraw; www.figdraw.com). PD-1, programmed cell death protein 1; PD-L1, programmed cell death ligand 1; CTLA-4, cytotoxic T lymphocyte antigen-4; IDO1, indoleamine 2,3-dioxygenase 1; ATP, adenosine triphosphate; AMP, adenosine monophosphate; MDSC, myeloid-derived suppressor cells; FOXP3, Forkhead box P3; FAO, fatty acid oxidation.

Studies have shown that the occurrence and progression of TC are closely associated with metabolic reprogramming of amino acids, glucose, and lipids in cancer cells. This metabolic reprogramming enhances the nutritional competitive ability of TC cells in an energy-limited microenvironment, creating favorable conditions for tumor growth (8). The metabolic processes produce a variety of products (such as lactate, adenosine, kynurenine, ketone bodies, ROS, and potassium ions), which contribute to a hypoxic, acidic, and nutrient-poor environment. These conditions exert inhibitory effects on neighboring immune cells, thereby altering tumor cell invasion, dissemination, and drug resistance, ultimately enabling TC to evade the immune system and achieve immune escape (91) (**Figure 3**).

### Amino acid metabolism

Glutamine regulates the level of ROS in the TME of PTC and promotes tumor cell development by regulating MAPK and PI3K/Akt pathways (92). In addition, α-ketoglutaric acid (α-KG), produced by glutamine metabolism, is abundant in human TC tissues and may promote the differentiation of macrophages into the M2 phenotype, enhancing the proliferation, migration, invasion, and stemness characteristics of TC cells (20). The glutaminase inhibitor 6-diaza-5-oxo-l-norleucine (DON) enhances the activity of CD8^+^ T cells in the TME and reduces the proliferation and metastasis of TC *in vivo* (93).

Serine hydroxymethyltransferase 2 (SHMT2) catalyzes the conversion of serine to glycine. In TC, high expression of SHMT2 leads to the secretion of immunosuppressive factors, including TGF-β, IL-6, and IDO (94). Phosphoglycerate dehydrogenase (PHGDH) is a key enzyme involved in serine synthesis. Inhibition of PHGDH by NCT503 activates AMP-activated protein kinase (AMPK), inhibits the mammalian target of rapamycin 1 (mTOR1) signaling pathway, upregulates p53 and p21 protein levels, and suppresses the proliferation and colony formation ability of TC cells (95).

Aminoxyacetic acid (AOA) is a transaminase inhibitor. Jiang et al. reported that AOA can inhibit TC tumor growth via ROS-mediated PI3K/AKT/mTOR and Wnt/β-catenin pathways (96).

### Glucose metabolism

In nonmedullary thyroid carcinoma, TAMs activate glucose metabolic reprogramming via the PI3K/Akt/mTOR pathway (97). These reprogrammed TAMs enhance proctumor effects by secreting TNF-α and VEGF, creating a protumor environment associated with TC progression (98).

During immune cell proliferation, glycolysis supports the proliferation of Tregs by providing energy in the form of adenosine triphosphate (ATP) and nicotinamide adenine dinucleotide (NADH). Glycolysis also regulates Treg activity by influencing the expression of specific variants in the transcriptional regulator, FOXP3 exon 2. Tregs have been associated with more aggressive forms of PTC (99).

When used in combination with a ketogenic diet, 3-bromopyruvic acid (3-BP) can effectively block the growth, migration, and invasion of TC cells by inhibiting glycolysis. 3-BP also significantly prolonged survival in a mouse model of ATC (100). In DTC, metformin exerts an indirect antitumor effect by inhibiting the activities of key regulators of glycolysis, including lactate dehydrogenase A (LDHA), phosphatidylinositol kinase 2 (PKM2), glutamate transporter 1 (GLUT1), and hydroxykinase 2 (HK2), while increasing the expression of isocitrate dehydrogenase 1 (IDH1) (101). In addition, metformin combined with the glycolytic inhibitor 2-deoxyglucose (2-DG) effectively inhibited IFN-γ production and cell proliferation in activated human CD4^+^ T cells (102).

In PTC, LDHA promotes the conversion of pyruvate to lactate, and in BRAF V600E mutant tumor cells, high expression of LDHA affects tumor invasiveness through the STAT3/LINC00671/LDHA axis (103). However, overexpression of LDHB interferes with aerobic glycolysis, proliferation, migration, and invasion (104). In MTC, lactate inhibits the production of major histocompatibility complex class II molecules (MHC-II) in antigen-presenting cells within the TME by activating G protein-coupled receptor 81 (GPR81), enabling MTC cells to evade immune attack and worsening patient prognosis (105).

### Lipid metabolism

Lipid metabolism in cancer cells is regulated not only by intracellular carcinogenic signals but also by various cytokines and growth factors (106). Targeting adipokines, such as adiponectin (APN) and leptin, as well as modulating cholesterol metabolism in immune cells of TC patients, represents a promising antitumor strategy to improve prognosis (107).

Cholesterol activates macrophages through disease-specific cytolyins (CDCs) and mediates EMT in human TC cells via the IL-8–Akt–SLUG, promoting extracellular migration (108). Statins can reduce cholesterol levels and inhibit TC cell proliferation, while also promoting apoptosis and differentiation. High VEGF expression in TC has been closely associated with advanced tumors and distant metastasis (109). Statins also exert antiangiogenic effects and, when combined with an anti-PD-1 antibody, simvastatin and lovastatin can slow tumor growth, improve survival, and promote TAM polarization toward a more M1-like phenotype (110).

APN receptor agonist AdipoAI reduces the production of proinflammatory cytokines in macrophages by inhibiting the activation of the NF-κB, MAPK, and c-Maf pathways. Mice treated with AdipoAI showed decreased messenger RNA (mRNA) expression levels of IL-1β and IL-6 (111), which affected the activation of the IL-6/JAK1/STAT3 pathway and promoted the occurrence and metastasis of TC (112).

Leptin is a protein-like hormone secreted by adipose tissue. It promotes the development of Th17 cells by upregulating the expression of RORγt gene, while inhibiting the differentiation and proliferation of Tregs. An increased number of Tregs is associated with greater aggressiveness of PTC (113).

According to a recent study by Lin et al., apolipoprotein E (APOE) can increase the infiltration of B cells, CD8^+^ T cells, and other immune cells in PTC (114). Additionally, Huang et al. reported that fat mass and obesity-associated protein (FTO) inhibits APOE expression through IGF2BP2-mediated m6A modification and may suppress glycolytic metabolism in PTC by modulating IL-6/JAK2/STAT3 signaling pathway, thereby restricting tumor growth (115).

## BRAF V600E mutation

The BRAF V600E mutation is the most common gene alteration in TC. Studies have reported that 36% of patients with PTC and 10%–50% of patients with ATC carry this mutation, which is associated with a poorer prognosis (116). BRAF V600E promotes TC progression, in part, by increasing the number of MDSCs (51).

This mutation is closely associated with elevated expression of immunosuppressive regulators in PTC cells. Expression levels of CTLA-4 and PD-L1 are inversely correlated with the thyroid differentiation score (TDS), with this relationship being more pronounced in tumors harboring thee BRAF V600E mutation (117). Furthermore, BRAF V600E-mutated tumors exhibit higher PD-1 expression compared with BRAF V600E–wild-type tumors (53% *vs*. 12.5%).

Patients with upregulated IDO, ARG-1, and PD-L1 were found to have a lower intratumoral CD8^+^/Foxp3^+^ cell ratio, which is strongly associated with the BRAF V600E mutation (118). In addition, in PTC patients with BRAF V600E mutation, the density of TILs increases (119), while the infiltration of CD8^+^ T-cell infiltration decreases (120). These changes ultimately promote PTC progression and negatively affect patient prognosis.

## Immunologic classification

There are several immunologic classification methods for TC based on tumor immune status.

Large-scale immunogenomic analysis based of TCGA data across cancer types have identified six immune subtypes: wound healing, IFN-γ dominant, inflammatory, lymphocyte-depleted, immunologically quiet, and TGF-β dominant. These subtypes are characterized by differences in macrophage or lymphocyte signatures, Th1/Th2 cell ratio, intratumoral heterogeneity, aneuploidy, neoantigen load, overall cell proliferation, expression of immunomodulatory genes, and prognosis. PTC is classified as an inflammatory subtype, characterized by a balanced ratio of macrophage-to-lymphocyte ratio (MLR), low intratumor heterogeneity, low aneuploidy, minimal somatic copy number variation, weak cell proliferation, and high expression of the Th17 differentiation-related genes (121).

Other studies have shown that TC can be classified into four types (hot, excluded, immunosuppressed, and cold) based on immune cell density and immune score. Hot tumors are characterized by substantial infiltration of T cells and CTLs in both the tumor center (CT) and invasive margin (IM) regions, high immune scores, and suppression of T-cell function due to activation of immune checkpoints such as PD-1, CTLA-4, TIM-3, and LAG-3. Excluded tumors showed no T-cell infiltration in the CT region, with moderate immune scores in the IM region; they are also associated with carcinogenesis activation, abnormal vascular/skeletal matrix proliferation, and hypoxia. Immunosuppressed tumors show moderate T-cell and CTL infiltration and immune scores, and contain T-cell checkpoints (PD-1, CTLA-4, TIM-3, and LAG-3), immunosuppressive cells (MDSCs and Tregs), and inhibitory cytokines (TGF-β, IL-10, and VEGF). Cold tumors had the lowest immune score, lack T-cell infiltration in CT and IM regions, exhibit low tumor mutation burden, poor antigen presentation, and T-cell insensitivity (122).

Another study further subclassified TC into ATC- and PDTC-like subtypes based on NGS and immunoscore data for CD3^+^ cell density. ATC-like tumors exhibit high levels of T-cell infiltration, chemokines (CCL2, CCL3, CCL4, CCL5, CXCL9, and CXCL10), and immune checkpoint expression. PDTC-like tumors are characterized by T-cell infiltration but show lower levels of chemokines and immune checkpoint expression (58).

## Immunotherapy strategies

### Cellular treatment strategies

A large number of preclinical and clinical studies related to immune cells and CAFs have been conducted to explore their potential as therapeutic targets for TC.

### Cancer vaccines

Tumor-specific antigens (TSAs) are highly immunogenic molecules expressed exclusively in tumor cells and are considered ideal targets for cancer vaccines (123). In TC, molecular mutations in signaling pathways such as BRAF, RAS, RET, and PTEN give rise to new antigens, including BRAF V600E, KRAS, and PI3K, which represent potential targets for tumor-specific immunotherapy (124). Compared with other TC subtypes, ATC expresses a higher abundance of neoantigens (125). As ATC typically harbors a high mutational burden, it presents more novel antigens suitable for tumor vaccines, suggesting that vaccine-based immunotherapy may be particularly effective in ATC (126).

MTC can secrete carcinoembryonic antigen (CEA). In a clinical trial (NCT01856920) for metastatic MTC, researchers evaluated a heat-killed yeast CEA vaccine, which demonstrated potential therapeutic application (127). Induction of systemic antitumor immunity using oncolytic virus (OV) vaccines has also been explored for thyroid cancer treatment of TC. Among them, the OV dl922–947 not only inhibited tumor growth in an ATC mouse model but also promoted the polarization of M2 TAMs into M1 TAMs (128).

## Adoptive cell therapy

Adoptive cell therapy (ACT) is an immunotherapy that isolates immunocompetent cells from cancer. The first approach involves transplanting immune cells, expanded *ex vivo*, into a patient, where they can directly attack the tumor or stimulate an immune system response. In patients with advanced PTC, FTC, and MTC, ACT using DCs pulsed with tumor lysates has successfully alleviated symptoms (129).

A second approach involves engineering T cells to become chimeric antigen receptor-T (CAR-T) cells, which specifically recognize and kill cancer cells. In selecting CAR-T targets for TC, tissue-specific antigens expressed on normal and cancerous thyroid cells can be screened, such as the thyroid-stimulating hormone receptor (TSHR) and glial-cell-line-derived neurotrophic factor (GDNF) family receptor α 4 (GFRα4). In addition, nonspecific targets, including CEA, intercellular adhesion molecule-1 (ICAM-1), and immune checkpoint proteins such as CD26 (B7-H3), are also major research directions of CAR-T cell therapy in TC.

### TSHR

TSHR is expressed on the basolateral membrane of thyroid follicular cells. The development and differentiation of thyroid cells and their hormone secretion and release are regulated through the interaction between TSHR and thyroid-stimulating hormone. Previous studies have confirmed that TSHR is present in both healthy thyroid tissues and thyroid tumors (130).

Li et al. developed a kind of CAR-T cell with two costimulatory domains targeting TSHR as a tumor-associated antigen. An *in vivo* experiment using a DTC xenograft mouse model showed that these CAR-T cells can effectively reduce tumor volume (131). Additionally, a second-generation CAR-T cell therapy targeting TSHR and CD19 (TSHR^+^CD19 CAR-T) in a patient with relapsed and refractory TC achieved a partial response (PR) at 3 months and was well tolerated, suggesting that CAR-T cell therapy offers a promising new direction for the treatment of TC (132).

### GFRα4

GFRα4 is a member of the GDNF receptor family, and its α subtype is expressed in both normal and malignant human MTC cells. Given the limited expression profile of GFRα4 in MTC, it may serve as a potential specific antigen target for CAR-T cell therapy.

Bhoj et al. developed CAR-T cells incorporating CD3ζ and CD137 costimulatory domains and demonstrated that these GFRα4-specific CAR-T cells eliminated tumors in an MTC xenograft mouse model. Antitumor activity was accompanied by a T-cell expansion phenomenon, suggesting that these cells represent a novel approach to immunotherapy for MTC (133). The University of Pennsylvania has initiated a phase I clinical trial to assess the safety and feasibility of GFRα4 CAR-T cells in the treatment of recurrent or metastatic MTC (NCT04877613).

### CEA

CEA is a tumor-associated antigen, and its expression level is positively correlated with cancer malignancy. Erickson et al. reported the design and synthesis of CEA-specific CAR-T cells for the treatment of MTC. Both *in vitro* and mouse experiments confirmed that CEA-specific CAR-T cells exerted a significant cytotoxic effect on CEA-positive MTC cells (134).

### ICAM-1

ICAM-1 is a cell surface glycoprotein and adhesion receptor involved in key physiological processes including signal transduction, immune responses, and inflammation. Studies have shown that ICAM-1 expression is elevated in TC and that it participates in tumor-related processes such as invasion and metastasis (135).

Min et al. were the first to report a CAR-T cell therapy targeting ICAM-1 for TC. *In vitro* experiments demonstrated that ICAM-1 CAR-T cells exerted significant cytotoxic effects on PTC and ATC cell lines expressing ICAM-1. In mice with systemic ATC, ICAM-1 CAR-T cells effectively inhibited tumor growth, maintained long-lasting antitumor activity, and significantly prolonged survival (136). AffyImmune Therapeutics Inc. has initiated a phase I clinical trial of ICAM-1 CAR-T cells for patients with recurrent/refractory PDTC and BRAF wild-type ATC, aiming to evaluate the safety and tolerability of its product AIC100 (NCT04420754). Preclinical trial reports indicate that AIC100 exhibits robust expansion in PTC patients, demonstrates an excellent safety profile, and effectively eliminates tumors (137).

### B7-H3

As a B7 family immune checkpoint protein, B7-H3 plays a key role in cancer immune escape, tumor metastasis, angiogenesis, and treatment resistance. Studies have indicated that targeting B7-H3 may be a promising strategy for MTC immunotherapy (70).

The B7-H3-targeted CAR-T cells designed by Duan et al. were constructed by linking the antigen-binding fragment (Fab) to the intracellular signaling domain of the native T-cell receptor (TCR), resulting in novel Fab CAR-T cells. Fab CAR-T cells can recognize tumor antigens independently of MGC and simulate the natural activation process of endogenous TCR, effectively addressing premature T-cell exhaustion. Moreover, in the TC, Fab CAR-T cells can induce cytotoxic effects (138).

### CAFs

CAFs are ideal therapeutic targets. They exhibit genetic stability and play a crucial role in maintaining the ECM framework of cancer cells. Moreover, they constitute a primary barrier that cancer cells exploit to resist anticancer drugs.

1. Targeting the matrix barrier to facilitate effective drug transport. TGF-β, matrix metalloproteinases (MMPs), and the Hedgehog pathway are the main therapeutic targets in the matrix barrier. Inhibition of TGF-β1 effectively suppresses the proliferation, migration, and invasion of ATC cells (8505C and/or SW1736) (139). The MMP inhibitor BB94 significantly reduces the migratory and invasive abilities of ATC cells (140). Similarly, the Hedgehog inhibitor cyclopamine decreases the expression of cancer stem cell-related transcription factors, including B lymphoma Mo-MLV insertion region 1 homolog (BMI1) and SRY-Box transcription factor 2 (SOX2), thereby inhibiting the growth of tumors derived from cancer stem cell xenograft models (141).
2. The potential targets of inhibiting CAFs secreted molecules that activate cancer cells include CTGF, HGF-c-MET pathway, and the CXCR4–CXCL12 axis. c-MET inhibitors (tevantinib and crizotinib) have been shown to inhibit cell proliferation in 50% of TC cell lines (142). By blocking the NF-κB signaling pathway, BAY11-7082, an inhibitor of the CXCR4–CXCL12 axis, successfully inhibited CXCL12–CXCR4-induced cell migration, invasion, and EMT in the PTC cell line (B-CPAP) (143).
3. Blockade of ECM components to reduce adhesion-induced signaling, with key targets including β-integrin and CD44. The TC-specific integrin-linked kinase inhibitor T315, even at extremely low concentrations, can inhibit cancer cell migration and show cytotoxic effects (144).

## Immune checkpoint inhibitors

Immune checkpoint blockade has markedly transformed cancer immunotherapy. Immune checkpoints act as key immunomodulatory elements, maintaining immune homeostasis and preventing autoimmunity (145). At present, monoclonal antibodies targeting PD-1, PD-L1, and CTLA-4 are the most widely studied immune checkpoint inhibitors in TC. Multiple phase III clinical trials have evaluated the efficacy and safety of anti-PD-1, anti-PD-L1, and anti-CTLA-4 antibodies, either as monotherapy or in combination with other immunotherapies, targeted therapies, or chemotherapy.

### Single immune checkpoint inhibitor

Recently, Oh et al. enrolled 103 patients with PTC or FTC who had received pembrolizumab (200 mg, q3w, IV) (**Table 1**). These patients experienced had experienced disease progression, treatment intolerance, and/or unresectable disease after at least one prior therapy. The ORR was 6.8% (95% confidence interval [CI], 2.8%–13.5%). Among patients with a PD-L1 combined positive score (CPS) ≥ 1 (*n* = 46), ORR was 8.7% (95% CI, 2.4%–20.8%), whereas among patients with PD-L1 CPS < 1 (*n* = 53), ORR was 5.7% (95% CI, 1.2%–15.7%) (146).

**Table 1:** Clinical studies for ICIs in thyroid cancers.

Treatments	Year	Country	ClinicalTrials.gov ID	Study	Dosage regimens	Patients	Enrollment (*n*)	Primary outcomes	AEs	Reference
Pembrolizumab (anti-PD-1)	2019	Worldwide	KEYNOTE-028 (NCT02054806)	Nonrandomized, phase Ib study	10 mg/kg, q2w, IV	Advanced DTC expressing PD-L1	22	ORR: 9%, mPFS: 7 months, mOS: not reached	Diarrhea, fatigue, colitis (grade 3)	(163)
Pembrolizumab (anti-PD-1)	2023	Worldwide	KEYNOTE-158 (NCT02628067)	Phase II	200 mg, q3w, IV	Advanced PTC/FTC failure of or intolerance to prior therapy	103	ORR: 6.8%, mOS: 34.5 months, mPFS: 4.2 months	69.9%, grades 3–5: 14.6%	(146)
Spartalizumab (anti-PD-1)	2020	Worldwide	NCT02404441	Phase II	400 mg, q4w, IV	Locally advanced and/or metastatic ATC	42	ORR: 19%	Diarrhea (12%), pruritus (12%), fatigue (7%), pyrexia (7%)	(147)
Pembrolizumab (anti-PD-1)/Nivolumab (anti-PD-1)	2022	USA		Single-center study		Locally advanced or metastatic unresectable ATC	13	ORR: 16%, mPFS: 1.9 months, mOS: 4.4 months	46%, grade 3 or higher: 15%, one patient with grade 5 immune checkpoint-related thyroiditis	(148)
AcSé pembrolizumab (anti-PD-1)	2021	France	NCT03012620	Phase II, nonrandomized parallel arm, open-label, multicentric study	200 mg, IV, q21d	Progressive RAIR TC	43	DTC: PR: 11.1%, SD: 18.5%, mPFS: 2.6 months, mOS: 12.7 months; ATC: PR: 18.8%, SD: 6.2%, mPFS: 2.3 months, mOS: 3.6 months	Grades 1–2: 9, grade 3: 20, grade 4: 1 (sepsis)	(149)
Spartalizumab (PDR001) (anti-PD-1)	2018	Worldwide	NCT02404441	Phase I/II, open-label, dose escalation/expansion study	400 mg, q4w, IV	ATC	26	ORR: 17% (RECIST 1.1), 20% (irRC); DCR: 27% (RECIST 1.1), 33%(irRC)	G3/G4: anemia, hypophosphatemia, lymphopenia, and oncologic complication	(150)
Nivolumab (anti-PD-1) + ipilimumab (anti-CTLA-4)	2020	USA	NCT03246958	Phase II	Nivolumab (3 mg/kg, q2w, IV) + ipilimumab (1 mg/kg, q6w, IV)	RAIR DTC, ATC, MTC	49	ORR: DTC: 9.4%, ATC: 30%, MTC: 0	Grades 3–4: increased lipase (*n* = 8), increased serum amylase (*n* = 4)	(151)

ICIs, Immune checkpoint inhibitors; AEs, adverse events; PD-1, programmed cell death receptor 1; IV, intravenous injection; DTC, differentiated thyroid cancer; PD-L1, programmed death ligand 1; PTC, papillary thyroid carcinoma; FTC, follicular thyroid carcinoma; mOS, median overall survival; mPFS, median progression-free survival; ATC, anaplastic thyroid carcinoma; USA, the United States; RAIR, radioactive iodine refractory; TC, thyroid cancer; DCR, disease control rate; MTC, medullary TC; ORR, objective response rate; PR, partial response; SD, stable disease; CTLA4, cytotoxic T lymphocyte-associated protein 4.

Capdevila et al. conducted a phase I/II trial in patients with ATC treated with spatalizumab. A total of 42 patients with locally advanced and/or metastatic ATC were enrolled and received 400 mg intravenously every 4 weeks. The ORR according to RECIST 1.1 criteria was 19% (including a 7% complete response). All patients who achieved a complete response had BRAF–wild-type tumors. The percentage of patients with PD-L1-positive (29%) was significantly higher than that of PD-L1-negative patients (0%). The 1-year survival rate of PD-L1-positive patients was 52.1% (147).

Another study showed a 16% response rate with pembrolizumab or nivolumab in 13 patients with locally advanced or metastatic unresectable ATC, including two partial responses. The 1-year survival rate was 38% (148).

The phase II trial (NCT03012620) by Leboulleux et al. evaluated pembrolizumab in progressive RAIR TC. The results showed a median overall survival (mOS) of 12.7 months and a median progression-free survival (mPFS) of 2.6 months. At six months, the OS rate was 73.3%, PFS rate was 16.9% (149).

A study of 26 heavily pretreated patients with advanced ATC showed a response rate of 17% with the anti-PD-1 monoclonal antibody Spatalizumab, opening up a new approach to ATC immunotherapy (150).

Another phase II trial evaluated nivolumab plus ipilimumab in three cohorts: the RAIR DTC cohort (*n* = 32, with four PDTC patients) and two exploratory cohorts (10 ATC patients and seven MTC patients). The ORR was 9.4% in the DTC cohort, 30% in ATC, and 0% in MTC (151).

### Immune checkpoint inhibitors combined with tyrosine kinase inhibitors

ICIs combined with TKIs are the most promising treatment (**Table 2**). ICIs regulate the immune system and enhance its ability to attack tumor cells by weakening the inhibitory effect on T-cell function. Lenvatinib, on the other hand, retards tumor growth by blocking the VEGF or vascular endothelial growth factor receptor (VEGFR) pathway and reducing angiogenesis. In addition, VEGF inhibition can reverse the immunosuppressive phenomena in the TME by inhibiting Tregs, M2 macrophages, and MDSCs.

**Table 2:** Clinical studies for ICIs combined with TKIs in thyroid cancers.

Treatments	Year	Country	ClinicalTrials.gov ID	Study	Dosage regimens	Patients	Enrollment (*n*)	Primary outcomes	AEs	Reference
Pembrolizumab (anti-PD-1) + KI (kinase inhibitor)	2018	USA		Retrospective study	Pembrolizumab (anti-PD-1) + kinase inhibitor (KI)	ATC progression on their KI therapy	12	BOR: PR: 42%, SD: 33%, PD: 25%	Fatigue, anemia, and hypertension	(152)
Lenvatinib (TKI) + pembrolizumab (anti-PD-1)	2021	Germany		Retrospective study	Lenvatinib, 14–24 mg, PO + pembrolizumab, 200 mg, q3w, IV	ATC, PDTC	6 ATC pts and 2 PDTC pts	BOR: ATCs: 66% (CR: 67%, SD: 16%, PD: 16%); PDTCs: (PR: 100%); mPFS: 17.75 months, mOS: 18.5 months	Grade III/IV: 50%	(153)
Lenvatinib (TKI) + pembrolizumab (anti-PD-1)	2022	Germany	ATLEP trial	Prospective, phase II study	Lenvatinib, 20 mg, qd, PO + pembrolizumab, 200 mg, q3 w, IV	ATC, PDTC	27 ATC pts and 8 PDTC pts	ORR: 34.3% (PR: 12/35); BOR: ATC: PR: 51.9%, SD: 44.4%; PDTC: PR: 75%, SD: 25%; CBR: ATC: 96.3%, PDTC: 100%; mPFS: ATCs: 9.5 months, PDTCs: 20 months; mOS: ATCs: 10.25 months, PDTCs: not reached	Grade III/IV: hemorrhage after fistula development, autoimmune hepatitis, pulmonary embolism and infectious complications including aspergillus pneumonias	(154)
Lenvatinib (TKI) + pembrolizumab (anti-PD-1)	2020	USA	NCT02973997	Single-arm multicenter phase II international study	Lenvatinib, 20 mg, qd, PO + pembrolizumab, 200 mg, q3w, IV	RAIR progressive DTC	30	PR: 62%, SD: 35%; CBR: 97%, mPFS: not reached	Grade 3: 70%, grade 4: 10%, > grade 3: hypertension (47%), weight loss (13%), maculopapular rash (13%), leukopenia (7%), diarrhea (7%) and oral mucositis (7%)	(155)

ICIs, Immune checkpoint inhibitors; TKIs, target kinase inhibitors; PD-1, programmed cell death receptor 1; KI, kinase inhibitor; ATC, anaplastic thyroid carcinoma; BOR, best overall response; PR, Partial response; SD, stable disease; PD, progrssive disease; AEs, adverse events; USA, the United States; DTC, differentiated thyroid cancer; IV, intravenous injection; PDTC, poorly differentiated thyroid cancer; PO, peros; mOS, median overall survival; mPFS, median progression-free survival; CBR, clinical benefit rate; ORR, objective response rate; RAIR, radioactive iodine-refractory.

Iyer et al. conducted a retrospective analysis of 12 patients with ATC who received pembrolizumab combined with TKIs after disease progression. The results showed that five cases (42%) achieved a partial response (PR), four cases (33%) had stable disease (SD), and three cases (25%) experienced progressive disease (PD) (152).

Dierks et al. reported treatment with lenvatinib plus pembrolizumab in six patients with metastatic ATC and two patients with PDTC. Among the ATC patients, 66% achieved complete response (CR), 16% had SD, and 16% had PD after up to 40 months of treatment. Both PDTC patients achieved PR. Grade III/IV toxicity was observed in half of the patients (four patients). Interestingly, patients with the highest tumor mutational burden (TMB) or PD-L1 expression had the best response (153).

Based on these findings, the phase II ATLEP trial further evaluated lenvatinib plus pembrolizumab in 27 ATC patients and eight PDTC patients. The ORR at 3 months was 34.3% (12/35 patients had a PR), with a best ORR of 51.9% and a SD rate of 44.4% over 2 years of treatment. The clinical benefit rate (CBR) was 96.3%, mPFS was 9.5 months, and OS was 10.25 months. Notably, 25.9% of patients survived beyond 2 years (154).

The efficacy of lenvatinib in combination with pembrolizumab was further validated in a single-arm phase II trial in patients with RAIR DTC. Preliminary results, presented at the 2020 ASCO annual meeting, showed that among 29 evaluable patients, the combination therapy achieved an ORR of 62% (no CR) and a 12-month PFS of 74% (155).

### Immune checkpoint inhibitors combined with chemoradiotherapy

Radiotherapy uses high-energy X-rays to shrink tumors and kill cancer cells. It is also commonly applied in the treatment of TC, such as iodine-131 therapy for DTC. In addition, radiotherapy can achieve more targeted effects by focusing on molecules preferentially expressed by cancer cells. This approach is often used when residual cancer cells remain after thyroidectomy or when cancer has metastasized to other sites (156). In a trial of stereotactic body radiation therapy (SBRT) combined with either the CTLA-4 inhibitor trastuzumab or the PD-L1 inhibitor dulazumab for metastatic ATC, the mOS was 14.5 weeks, with only one patient surviving more than a year (**Table 3**) (157).

**Table 3:** Clinical studies for ICIs combined with chemoradiotherapy in thyroid cancers.

Treatments	Year	Country	ClinicalTrials.gov ID	Study	Dosage regimens	Patients	Enrollment (*n*)	Primary outcomes	Reference
Durvalumab (MEDI4736) (anti-PD-L1) + tremelimumab (anti-CTLA4) + image-guided SBRT	2022	USA	NCT03122496	Pilot, phase I study	Tremelimumab, 75 mg, q4w, IV + durvalumab, 1,500 mg, q4w, IV + SBRT (9 Gy × 3 fractions)	Metastatic ATC	12	mOS: 14.5 weeks	(157)
Pembrolizumab (anti-PD-1) + chemoradiotherapy	2019	Minnesota, USA		Investigator-initiated therapeutic phase II trial	Pembrolizumab, 200 mg, q3w, IV + chemoradiotherapy (docetaxel/doxorubicin, 20 mg/m^2^, qw, IV + volumetric modulated arc therapy)	ATC	3	6mOS: 0	(158)

ICIs, immune checkpoint inhibitors; PD-1, programmed cell death receptor 1; CTLA4, cytotoxic T-lymphocyte-associated protein 4; SBRT, stereotactic body radiotherapy; IV, intravenous injection; ATC, anaplastic thyroid carcinoma; mOS, median overall survival; USA, the United States; 6mOS, overall survival rate at 6 months.

Docetaxel and doxorubicin hydrochloride are commonly used chemotherapeutic drugs. They inhibit tumor growth through a variety of mechanisms, including cell killing, suppression of cell division, and prevention of metastasis. In a phase II trial, Chintakuntlawar et al. evaluated three patients phase II trial evaluated three patients with Pembrolizumab combined with chemoradiotherapy, and although some patients had good initial responses, all patients died within 6 months of treatment initiation (158).

### Immune checkpoint inhibitors combined with BRAF inhibitors

BRAF inhibitor (BRAFi) therapy can precisely kill TC cells harboring the BRAF V600E oncogene. Multiple selective BRAFs (dabrafenib/vemurafenib) have been successfully developed (**Table 4**). However, the recurrence-free survival (RFS) of BRAF-mutant TC patients is typically limited to 6–7 months (159). Activation of the MEK/MAPK pathway is observed in most patients who develop resistance (160). The limited efficacy of BRAFi monotherapy is closely associated with persistent high tumor PD-L1 expression levels. In comparison, combining BRAFi with ICIs enhances antitumor activity and significantly prolongs survival (62).

**Table 4:** Clinical studies for ICIs combined with BRAFi in thyroid cancers.

Treatments	Year	Country	ClinicalTrials.gov ID	Study	Dosage regimens	Patients	Enrollment (*n*)	Primary outcomes	Reference
Atezolizumab (anti-PD-L1) + targeted therapy	2020	USA	NCT03181100	Prospective, single-center trial	Cohort I: vemurafenib (BRAFi), bid, PO + cobimetinib (MEKi), qd, PO + atezolizumab (Anti-PD-L1), q14d, IV Cohort II: cobimetinib, qd, PO + atezolizumab, q14d, IV Cohort III: atezolizumab, q21d, IV + bevacizumab (VEGFi), q21d, IV Cohort IV: nab-paclitaxel, q7d, IV + atezolizumab, q21d, IV	ATC	43	OS: 18.23 months	(161)
Dabrafenib (BRAFi) + trametinib (MEKi) followed by surgical resection and adjuvant chemoradiation	2019	Texas, USA			Dabrafenib, 150 mg, bid, PO + trametinib, 2 mg, qd, PO + radiotherapy (60 Gy in 30 fractions) + chemotherapy (paclitaxel and carboplatin, or cisplatin alone)	BRAFV600E-mutated ATC	6	6mOS:100%, 12mOS: 83% Locoregional control rate: 100%	(162)

ICIs, immune checkpoint inhibitors; BRAFi, BRAF inhibition; MEKi, MEK inhibition; PD-L1, programmed death ligand 1; PO, peros; IV, intravenous injection; VEGFi, VEGF inhibition; ATC, anaplastic thyroid carcinoma; OS, overall survival; USA, the United States; 6mOS, overall survival rate at 6 months; 12mOS, overall survival rate at 12 months.

A prospective multiarm trial involved three cohorts: patients with BRAF mutations received the anti-PD-L1 antibody atezolizumab plus vemurafenib or cobitinib (cohort 1); patients with RAS and NF1 mutations received cobitinib alone (cohort 2), and patients without mutations were treated with bevacizumab (cohort 3). Cohort 3 was closed early due to poor efficacy. The mOS for the three groups was 18.23 months, 6.21 months, and not reached, respectively. The response rate was 71% in cohort 1 and 7% in cohort 2. Based on the above findings, the combination of ICIs and targeted therapy shows great potential for the treatment of ATC patients with specific molecular alterations and is expected to significantly improve the prognosis and survival rate of ATC patients (161).

A recent study described six patients with BRAF-mutated ATC who received sequential treatment with dafinib and trametinib, surgery, and adjuvant chemoradiotherapy, three of these patients also received pembrolizumab. The OS rate at 6 months was 100%, and the 1-year survival rate was 83% (162).

In addition, we have summarized ongoing clinical trials that have not yet reached conclusions (**Supplementary Table**). These studies are expected to provide valuable guidance for future clinical management.

## Conclusion

In recent years, ICIs have been explored as a therapeutic strategy to restore antitumor T-cell function. In addition, multiple immune components associated with TC development provide novel therapeutic opportunities, including NK cell immunotherapy, DC vaccines, and blockade of M2 TAMs. ACT using DCs and CAR-T cells has demonstrated efficacy in patients with advanced PTC. Moreover, combining ICIs with VEGF inhibitors, radiotherapy, chemotherapy, and BRAFi can achieve improved therapeutic efficacy and is expected to offer additional clinical value for the treatment of PTC. In addition, studies in immunometabolism have shown that immune cell function is influenced by nutrient availability and tumor cell metabolic reprogramming, representing another important direction in immunotherapy research. A deeper understanding of tumor–immune system interactions is therefore crucial for identifying new molecular targets, developing innovative immunotherapeutic strategies, and discovering novel biomarkers that may enhance the efficacy of immunotherapy in TC.

## Future perspectives

Immune cells and molecules in the TME play a key role in the progression of TC by regulating the body’s immune response to cancer. Despite the promising prognosis of TC, the treatment of patients with advanced/metastatic DTC and ATC remains challenging, as existing targeted therapies are not curative, and many patients with advanced TC will eventually deteriorate due to acquired drug resistance. However, the underlying immune mechanisms remain unclear. Based on these research advances, the development of novel immune biomarkers for assessing disease severity and progression, as well as personalized therapies aimed at enhancing antitumor immune responses through innovative immune modulators, is anticipated to effectively address treatment failure and/or immunotherapy-related drug resistance.
