# Supplementary material for: New insights into the tumor immune microenvironment and immunotherapy of thyroid cancer
Source: Front Immunol. 2026 Jan 23;17:1699500. doi: 10.3389/fimmu.2026.1699500 (PMC12876178; doi:10.3389/fimmu.2026.1699500)
Supplement: Supplementary file 1 [file DataSheet1.docx]

Supplementary Table 1 Clinical trials for ICIs in thyroid cancers.

| Treatments | ClinicalTrials.gov ID | Study phase | Study type | Patients | Enrollment (Actual),n | Dosage regimens | Primary outcomes | Country | Trial status | Study completion (Actual) |
| --- | --- | --- | --- | --- | --- | --- | --- | --- | --- | --- |
| Pembrolizumab (anti-PD-1) | NCT03072160 | Phase 2 | Interventional | Recurrent or metastatic MTC | 17 | Pembrolizumab,200mg, q3w, IV | PR, CR | USA | Completed | 2019-11-22 |
| Pembrolizumab (anti-PD-1) | NCT05119296 | Phase 2 | Interventional | Metastatic or locally advanced ATC | 20 | pembrolizumab, 200mg, q3w, IV | ORR | USA | Recruiting | 2026-11 |
| Pembrolizumab (anti-PD-1) | NCT02688608 | Phase 2 | Interventional | ATC | 6 | pembrolizumab , 200mg, q3w, IV | ORR | USA | Completed | 2021-10 |
| Durvalumab (anti-PD-L1) + Tremelimumab (anti-CTLA4) | NCT03753919 | Phase 2 | Interventional | Progressive, refractory advanced TC | 79 | Durvalumab, 1500mg, q4w, IV + tremelimumab, 75 mg, q4w, IV | 6mPFS, 6mOS | Spain | Terminated | 2024-11-08 |
| Vudalimab (XmAb2071) (anti-CTLA4 & PD-1) | NCT05453799 | Phase 2 | Interventional | Locally advanced or metastatic ATC or Hurthle Cell TC | 54 | Vudalimab, q14d, IV | ORR | USA | Unknown status | 2024-07-15 |

Abbreviations: ICIs, Immune checkpoint inhibitors; MTC, Medullary thyroid cancer; USA, the United States; IV, Intravenous injection; PR, Partial response; CR, Complete response; ATC, Anaplastic thyroid cancer; ORR, Objective response rate; TC, Thyroid cancer; 6mPFS, Progression-free survival rate at 6 months; 6mOS, Overall survival rate at 6 months; CTLA4, Cytotoxic T-lymphocyte-associated protein 4; PD-1, Programmed cell death receptor 1.

Supplementary Table 2 Clinical trials for ICIs combined with TKIs in thyroid cancers.

| Treatments | ClinicalTrials.gov ID | Study phase | Study type | Patients | Enrollment (Actual),n | Dosage regimens | Primary outcomes | Country | Trial status | Study completion (Actual) |
| --- | --- | --- | --- | --- | --- | --- | --- | --- | --- | --- |
| Camrelizumab (anti-PD-1) + Apatinib (TKI) | NCT04612894 | Phase 2 | Interventional | Locally advanced TC | 31 | Apatinib, 250mg, qd, PO + Camrelizumab, 200mg, q2w, IV | ORR | China | Unknown status | 2023-12-31 |
| Lenvatinib (TKI) + Nivolumab (anti-PD-1) | NCT05696548 | Phase 2 | Interventional | ATC | 51 | Lenvatinib, 24mg, qd, PO + Nivolumab, 240mg, q2w, IV | Proportion of subjects with DLT, ORR | Japan | Active, not recruiting | 2026-12 |
| mTKI + anti-PD-1 | NCT04521348 | Phase 2 | Interventional | Advanced TC | 103 | mTKI + PD-1 | ORR | China | Completed | 2024-12-31 |
| Surufatinib (TKI) + Toripalimab (anti-PD-1) | NCT04524884 | Phase 2 | Interventional | Locally advanced TC | 10 | Surufatinib, 250mg, qd, PO + Toripalimab, 240mg, q21d, IV | ORR | China | Unknown status | 2022-09-30 |
| Camrelizumab (anti-PD-1) + Apatinib (TKI) | NCT04560127 | Phase 2 | Interventional | RAIR DTC | 20 | Apatinib, 250mg, qd, PO + Camrelizumab, 200mg, q2w, IV | ORR | China | Active, not recruiting | 2025-12-31 |
| Cabozantinib (TKI) + Nivolumab (anti-PD-1) + Ipilimumab (CaboNivoIpi) (anti-CTLA-4) | NCT03914300 | Phase 2 | Interventional | Advanced DTC | 9 | Cabozantinib, qd, PO + Nivolumab, IV + Ipilimumab, IV | ORR | USA | Active, not recruiting | 2025-12-17 |
| Lenvatinib (TKI) + Pembrolizumab (anti-PD-1) | NCT04171622 | Phase 2 | Interventional | Stage IVB locally advanced and unresectable or stage IVC metastatic ATC | 25 | Lenvatinib, qd, PO + Pembrolizumab, q21d, IV | OS, PFS, Tumor response | USA | Active, not recruiting | 2025-08-31 |

Abbreviations: ICIs, Immune checkpoint inhibitors; PD-1, Programmed cell death receptor 1; TKIs, Target kinase inhibitors; TC, Thyroid cancer; PO, peros; IV, Intravenous injection; ORR, Objective response rate; ATC, Anaplastic thyroid cancer; DLT, Dose-Limiting Toxicities; mTKI, multiple Target Kinase Inhibitor; RAIR, Radioactive iodine-refractory; DTC, Differentiated thyroid cancer; CTLA4, Cytotoxic T-lymphocyte-associated protein 4; USA, the United States; ATC, Anaplastic Thyroid Cancer; PFS, Progression-free survival; OS, Overall survival.

Supplementary Table 3 Clinical trials for ICIs combined with chemoradiotherapy in thyroid cancers.

| Treatments | ClinicalTrials.gov ID | Study phase | Study type | Patients | Enrollment (Actual),n | Dosage regimens | Primary outcomes | Country | Trial status | Study completion (Actual) |
| --- | --- | --- | --- | --- | --- | --- | --- | --- | --- | --- |
| Durvalumab (Medi4736) (anti-PD-L1) + RAI | NCT03215095 | Early Phase 1 | Interventional | RAI-avid, recurrent/metastatic TC | 11 | Durvalumab, 1500 mg, q4w, IV + RAI | Number of patients with DLTs | USA | Active, not recruiting | 2026-07 |
| Pembrolizumab (anti-PD-1) + IMRT | NCT05059470 | Phase 2 | Interventional | ATC | 6 | Pembrolizumab, q6w, IV + IMRT | mPFS | USA | Terminated | 2025-07-01 |
| Penpulimab (anti-PD-1) / Anlotinib (TKI) + RAI | NCT04952493 | Phase 2 | Interventional | Local advanced or metastatic differentiated TC | 42 | Cohort 1: Anlotinib + RAI  Cohort 2: RAI  Cohort 3: Penpulimab + RAI | ORR | China | Unknown status | 2024-07-20 |
| Anti-PD-1 + Anlotinib (TKI) + Multimodal Radiotherapy | NCT05659186 | Phase 2 | Interventional | Recurrent or metastatic ATC | 20 | Tislelizumab, 200mg, q3w, IV + Anlotinib, 12mg, qd, PO + Multimodal Radiotherapy | ORR | China | Recruiting | 2025-12-30 |
| Pembrolizumab (anti-PD-1) + Chemotherapy + Radiotherapy ± Surgery | NCT03211117 | Phase 2 | Interventional | ATC | 3 | Cohort A: Pembrolizumab 200 mg, q21d, IV + Surgery + docetaxel, q1w, IV + doxorubicin hydrochloride, q1w, IV + IMRT, once daily 5 days per week  Cohort B: Pembrolizumab 200 mg, q21d, IV + docetaxel, q1w, IV + doxorubicin hydrochloride, q1w, IV + IMRT, once daily 5 days per week | OSR | USA | Completed | 2019-03-28 |
| Atezolizumab (anti-PD-L1) + Chemotherapy | NCT03181100 | Phase 2 | Interventional | ATC or poorly DTC | 50 | Cohort I：vemurafenib (BRAFi), bid, PO + cobimetinib (MEKi), qd, PO + atezolizumab (Anti-PD-L1), q14d, IV  Cohort II：cobimetinib, qd, PO + atezolizumab, q14d, IV  Cohort III：atezolizumab, q21d, IV + bevacizumab (VEGFi), q21d, IV  Cohort IV: nab-paclitaxel, q7d, IV + atezolizumab, q21d, IV | OS | USA | Active, not recruiting | 2027-07-31 |

Abbreviations: ICIs, Immune checkpoint inhibitors; RAI, Radioiodine; RAI-avid, Radioactive iodine-avid; TC, Thyroid cancer; USA, the United States; IV, Intravenous injection; DLT, Dose-Limiting Toxicities; IMRT: Intensity-Modulated Radiation Therapy; ATC, Anaplastic thyroid cancer; mPFS, median Progression free survival; ORR, Objective response rate; PD-1, Programmed cell death receptor 1; PO, peros; OSR, Overall survival rate; DTC, Differentiated Thyroid Cancer; BRAFi, BRAF inhibition; MEKi, MEK inhibition; PD-L1: Programmed cell death-ligand 1; VEGFi, VEGF inhibition; OS,Overall survival; TKI, Target kinase inhibitor.

Supplementary Table 4 Clinical trials for ICIs combined with BRAFi in thyroid cancers.

| Treatments | ClinicalTrials.gov ID | Study phase | Study type | Patients | Enrollment (Actual),n | Dosage regimens | Primary outcomes | Country | Trial status | Study completion (Actual) |
| --- | --- | --- | --- | --- | --- | --- | --- | --- | --- | --- |
| Cemiplimab (anti-PD-1) + Dabrafenib (BRAFi) + Trametinib (MEKi) | NCT04238624 | Phase 2 | Interventional | BRAF-Mutant ATC | 16 | Cemiplimab + Dabrafenib, 150mg, bid, PO + Trametinib, 2mg, qd, PO | ORR | USA | Active, not recruiting | 2026-06-20 |
| Spartalizumab (PDR001) (anti-PD-1) + Trametinib (MEKi) / Dabrafenib (BRAFi) | NCT04544111 | Phase 2 | Interventional | RAIR TC | 19 | Cohort A (BRAF WT tumors): Trametinib, 2mg, qd, PO + Spartalizumab, 400mg, q4w, IV  Cohort B (BRAF Mutant tumors): Dabrafenib , 150 mg, bid, PO + Spartalizumab, 400mg, q4w, IV | ORR | USA | Active, not recruiting | 2025-09-02 |
| Dabrafenib (BRAFi) + Trametinib (MEKi) + Pembrolizumab (anti-PD-1) | NCT04675710 | Phase 2 | Interventional | BRAF-Mutated ATC | 30 | Dabrafenib, 150 mg, bid, PO + Trametinib, 2mg, qd, PO + Pembrolizumab, 200mg, q21d, IV | Complete gross surgical resection (R0 or R1 resection), OS | USA | Active, not recruiting | 2026-10-30 |
| Encorafenib (BRAFi) + Binimetinib (MEKi)±Nivolumab (anti-PD-1) | NCT04061980 | Phase 2 | Interventional | Metastatic RAIR BRAF V600 Mutant TC | 24 | Arm I: Encorafenib, qd, PO + Binimetinib, bid, PO  Arm II: Encorafenib, qd, PO + Binimetinib, bid, PO + Nivolumab, q28d, IV | ORR | USA | Active, not recruiting | 2027-10-30 |

Abbreviations: ICIs, Immune checkpoint inhibitors; PD-1, Programmed cell death receptor 1; BRAFi, BRAF inhibition; MEKi, MEK inhibition; RAIR, radioactive iodine-refractory; ATC, Anaplastic thyroid carcinoma; USA, the United States; PO, peros; ORR, Objective response rate; TC, Thyroid cancer; IV, Intravenous injection; OS, Overall survival.
